# Kikuchi-Fujimoto Disease: A Differential for When It is Not Systemic Lupus Erythematosus

**DOI:** 10.1155/2022/7709246

**Published:** 2022-08-09

**Authors:** Taskeen R. Kazmi, Emma L. Greear, Catherine A. Lavallee, Michael S. Stump, Adegbenga A. Bankole

**Affiliations:** ^1^Division of Rheumatology, Virginia Tech Carilion School of Medicine Carilion Clinic, 3 Riverside Circle Roanoke, Roanoke, VA 24016, USA; ^2^Virginia Tech Carilion School of Medicine, 2 Riverside Circle Roanoke, Roanoke, VA 24016, USA; ^3^Dominion Pathology Associates, Carilion Roanoke Memorial Hospital, 1906 Belleview Ave SE, Roanoke, VA 24014, USA

## Abstract

Kikuchi-Fujimoto disease (KFD) is a rare and benign disease process that is characterized by fever and lymphadenopathy that was first described in young Japanese women in the early 1970s. Knowledge of KFD is important as it can often mimic other causes of lymphadenopathy including systemic lupus erythematosus (SLE) or malignancies, and this can lead to invasive diagnostic testing and even treatments that can be avoided. The etiology and exact mechanism by which KFD develops is not fully understood at this time, but is thought to be an immune response of T cells and histiocytes to viral or bacterial infections. We present a 35-year-old African-American woman who was referred to the rheumatology clinic by our colleagues in the breast clinic with new onset right axillary lymphadenopathy and abnormal serologic testing with the suspicion of SLE after a malignancy had been ruled out.

## 1. Introduction

Kikuchi-Fujimoto disease (KFD) or Kikuchi histiocytic necrotizing lymphadenitis is a benign self-limited disorder that is characterized by fever and tender regional lymphadenopathy. It was first described in young Japanese women in 1972 [1, 2] but has since been seen across other ethnic groups. It remains a rare disease with most cases occurring in Asia. KFD most commonly affects adults under 40 years of age with a female preponderance [3]. In the United States, 75% of affected patients are Caucasian [4]. Pathogenesis remains unclear but it is thought to be an immune response of T cells and histiocytes due to viral infections [5]. Symptoms of KFD include tender cervical lymphadenopathy, low grade fever, leukopenia, headache, and fatigue [5–8]. KFD is often mistaken for other conditions such as lymphoma and systemic lupus erythematosus (SLE) [8], making its diagnosis clinically difficult. We present a case of a 35-year-old African-American female that was seen in southwest Virginia (USA) with unilateral axillary lymphadenopathy and a positive antinuclear antibody (ANA) with suspicions of SLE.

## 2. Case Presentation

A 35-year-old African-American woman was referred to the rheumatology clinic in 2019 prior to the onset of the current severe acute respiratory syndrome (SARS)/COVID-19 pandemic, for an evaluation of a low positive antinuclear antibody (ANA). The ANA was noted while she was being evaluated for unilateral axillary lymphadenopathy. She was seen in the breast clinic to evaluate a 1-week history of a lump in her right axilla. She did not notice obvious swelling but had felt a tender protuberance in her right armpit. She sought medical attention when she noted that the lump was increasing in size and becoming painful. The pain had no alleviating or aggravating factors. The breast lump did not change in size in relation to her menstrual cycle. She did not have mastalgia, nipple discharge, and other masses. Over the preceding months, she had noticed intermittent fevers, generalized weakness, fatigue, and paresthesia of extremities. She had no mouth ulcers, arthralgias, or rashes on sun-exposed skin. She noted no weight loss, night sweats, lymphadenopathy, hemoptysis, alopecia, joint swelling, skin tightening, cough, or shortness of breath.

She had diagnoses of Charcot-Marie-Tooth (CMT) disease, gastroesophageal reflux disease (GERD), and migraines. Her prior surgeries included appendectomy, cholecystectomy, hernia repair, and tubal ligation. At the time of presentation, she was being treated with bupropion, hydrocortisone cream, hydroxyzine, ketoconazole cream, probiotic complex, and sumatriptan. She had allergies to antibiotics including sulfonamide and amoxicillin-clavulanate. Her maternal great-aunt had breast cancer. The patient stopped smoking several years prior to this presentation.

In the rheumatology clinic, her blood pressure was 124/79 mmHg, pulse 95/min, temperature 97.4°F, and respiratory rate 14/min. Her extraocular movements, eyelids, and conjunctivae were normal. She had no alopecia, rashes, ulcers, or telangiectasias. She did not have other lymphadenopathy aside from her right axillary nodes. Her pulmonary and cardiovascular examination were normal, and she did not have muscle weakness or inflammatory joint findings. Her extremities were warm and well perfused without evidence of digital pallor or cyanosis. Her breast examination confirmed asymmetry with the left breast being slightly larger than the right but with no notable skin or nipple changes. There were no masses or lymphadenopathy in the right breast, but a 10 × 5 millimeter (mm) firm, tender, and mobile mass with smooth borders was palpated high in the right axilla. The mass was not attached to the skin or underlying musculature. The left breast and axilla showed no skin or nipple abnormalities.

Her laboratory tests revealed an ANA titer of 1 : 40 which is essentially a negative test but was flagged as positive in the lab system. Double stranded DNA (dsDNA) and anti-Ro/Sjogren's syndrome-related antigen A (SSA) antibodies were also noted to be elevated (Table 1 and Table 2).

Breast mammography revealed heterogeneously dense breasts, with a breast imaging reporting and data system (BI-RADS) score of 4, suggestive of a breast malignancy. Right breast ultrasound confirmed an abnormally enlarged lymph node in the right axilla with thickened cortex measuring 19 × 14 × 16 mm and a second enlarged lymph node in the right axilla measuring 21 × 9 × 14 mm.

**Figure 1:**
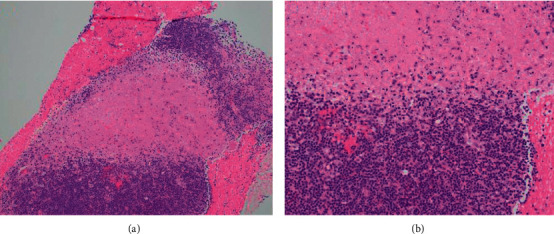
Hematoxylin and Eosin (H&E) statin ((A) 10x, (B) 20x) showing area of well-developed necrosis with a few surrounding histiocytes and without neutrophilic infiltrate.

**Figure 2:**
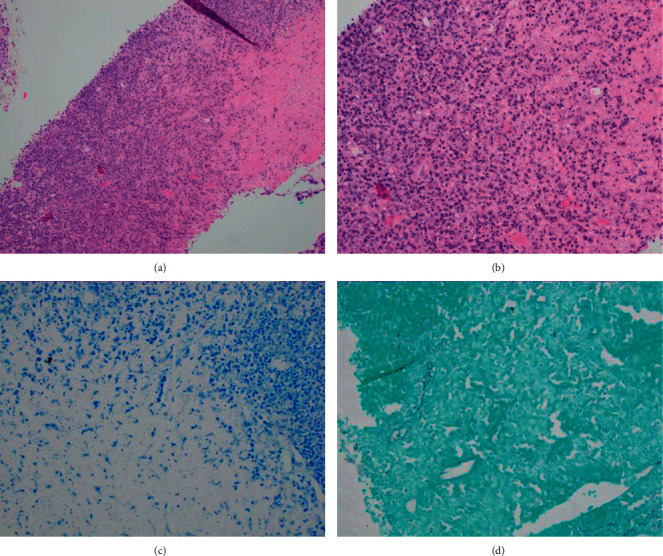
H&E statin ((A) 10x, (B) 20x): area of necrosis with more prominent ongoing necrosis and surrounding proliferation of histocytes and plasmacytoid dendritic cells. (C) Acid-fast bacteria (AFB) stain, 20x. (D) Gömöri methenamine silver stain (GMS) 20x. Negative staining for acid fast bacilli and fungal elements.

She did have a biopsy of the right axillary lymph node, and histopathology examination and flow cytometry were performed. The pathology confirmed the diagnosis of KFD based on the presence of proliferating histiocytic predominance necrosis without neutrophilic infiltration (Figures 1 and 2). The lymph node flow cytometry did not demonstrate B cell clonal expansion (Table 3).

## 3. Discussion

KFD was first described in the literature by Japanese pathologists Kikuchi and Fujimoto [1, 2]. Cases were initially reported in Japan and East Asia but have since been seen worldwide. KFD is predominantly seen in women younger than 40, but also occurs in men [6]. A fever is usually the first symptom in KFD and can be accompanied by upper respiratory symptoms (30–50%), weight loss, night sweats, nausea, vomiting, sore throat, arthralgia, splenomegaly, rash, and leukopenia (about 50%) [9]. Unilateral tender lymphadenopathy involving the jugular and posterior cervical lymph nodes is the most common clinical feature [7], although supraclavicular lymphadenopathy has also been noted [10]. Axillary lymphadenopathy has been reported, but is a rare manifestation of the disease [11, 12] and this was seen in our patient. The lymph nodes vary in size but are typically less than 4 centimeters (cm) [6]. In the United States, KFD is an uncommon cause of fever and lymphadenopathy, but should be considered in the right clinical setting.

The cause of KFD is still unknown. It is thought to be due to a viral/postviral infection based on the presence of a prodrome of upper respiratory symptoms [5, 9]. KFD also shares histopathologic features with some viral illnesses [5]. Although our case occurred before the current COVID-19 pandemic at present, there has been an increase in the reported cases of KFD. This may be related to the prevalence of COVID-19 during the current pandemic. A number of reports suggest that the rates of KFD in COVID-19 may be related to the cytokine storm [13]. The postviral nature of KFD is further buttressed by the reports of cases that have occurred following vaccination for COVID-19 [14]. The histological findings in both COVID-19 infections and its vaccination related KFD cases are similar with that of the classic KFD, including coagulative necrosis, apoptosis surrounded by lymphocytes, histiocytes, and karyorrhectic debris [14].

Other theories associate KFD to an autoimmune disease, specifically SLE. KFD is thought to be an SLE-like autoimmune condition induced by virus-infected transformed lymphocytes [15]. It is recommended that patients with KFD be assessed for SLE at diagnosis and have long-term follow-up with a rheumatologist to rule out development of SLE [4, 5]. In our patient, the ANA titer of 1 : 40 was so low that it was unlikely to be related to SLE based on the 2012 Systemic Lupus International Collaborating Clinics (SLICC) classification criteria [16]. A low-titer ANA does not usually yield a significantly positive dsDNA so the clinical utility of the dsDNA towards a diagnosis of SLE was also low. However, given the laboratory tests sent to the clinic as part of the referral, a full and thorough evaluation and workup was performed. The ANA, dsDNA, and SSA antibodies noted in our patient could be described clinically as false positives as the ANA test is very sensitive but not very specific for SLE [17]. Given these findings, our rheumatology team sought alternative diagnoses to SLE in our patient.

Other laboratory findings seen in KFD may include anemia and leukopenia with atypical lymphocytes in peripheral blood smear, although many patients have normal laboratory studies [6]. Elevations in erythrocyte sedimentation rate, lactate dehydrogenase, and liver function tests may also occur [6]. Excisional biopsy of the affected lymph node showing paracortical areas of coagulative necrosis, karryoherrectic debris, distortion of nodal architecture, histiocytic cellular infiltrates with predominance of CD8+ T-cells, absence of neutrophils, and few B cells confirms the diagnosis [15].

The course of KFD is self-limited [8], with no specific treatment required. Supportive therapy including analgesics, antipyretics, and rest is recommended. Patients with severe or persisting symptoms can be treated with glucocorticoids alone or in combination with hydroxychloroquine [5, 18]. Patients should be followed over time to assess for the development of SLE or recurrence of KFD [5]. An accurate diagnosis can prevent inappropriate treatment with antibiotic or anticancer therapy [11].

## 4. Conclusion

Although SLE should always be included in the differential when a young woman of African descent is being evaluated for lymphadenopathy, fever, and a positive ANA, there are several important diagnoses not to be missed including malignancy, especially lymphoma. In this case, the suspicion for SLE by the breast team led to a referral to rheumatology. With the ANA of titers of 1 : 80 and below, the likelihood of SLE is very low. Armed with this knowledge, a collaborative hunt for other causes ensued. This effort quickly resulted in the confirmation of KFD as the diagnosis in our patient. Unilateral axillary lymphadenopathy with fevers in an African-American patient is a rare presentation of KFD, and this presentation shares many features with lymphoma and infectious etiologies. Although the exact pathophysiology of KFD is not fully understood, an autoimmune and infectious workup should be considered in patients with suspected KFD. KFD typically resolves spontaneously within months, but patients should be monitored after resolution of symptoms as there are reports of recurrence of KFD as well as coexistence with and progression to SLE [19].

## Figures and Tables

**Table 1:** Component of complete blood count and metabolic panel.

Item	Reference range	Results
WBC	4.0–10.5 k/*μ*L	7.2
Hemoglobin	12.0–16.0 g/dL	13.4
Hematocrit	36–46%	39.2
Platelet count	130–400 K·*μ*L	246
Urea nitrogen	6–20 MG/DL	7
Creatinine	0.5–1.2 MG/DL	0.54
Alkaline phosphatase, serum	42–121 IU/L	58
AST	10–42 IU/L	18
ALT	10–60 IU/L	17

**Table 2:** Immunological tests.

Item	Reference range	Results
Anti-nuclear antibody (ANA) with HEp-2 substrate^*∗*^ Immunofluorescence assay (IFA)		Positive
ANA titer	<1 : 40	1 : 40
	1 : 40–1 : 80 weakly positive	
	> Or = to 1 : 160 result may be clinically significant	
ANA pattern		Nuclear, speckled
Anti-jo-1 Ab	<9 IU/mL	<1.0
Centromere B Ab	<9 IU/mL	<1.0
Complement C3	82–185 mg/dL	129
Complement C4	15–53 mg/dL	37
Rheumatoid factor	<14 IU/mL	<14
		**16**
	Negative ≤4 IU/mL	
Anti-DNA, native double strand IgG ELISA^*∗∗*^	Indeterminate 4–9 IU/mL	
	Positive ≥10 IU/mL	
Thyroid peroxidase (TPO) Ab	<9 IU/mL	<1
Ribosomal P protein Ab	<1.0 NEG AI	<1.0
Scleroderma (SCL-70)	<1.0 NEG AI	<1.0
Sjögren Ab SSA	<1.0 NEG AI	**3.3**
Sjogren Ab SSB	<1.0 NEG AI	<1.0
Anti-smith antibody	<1.0 NEG AI	<1.0
Sm/RNP	<1.0 NEG AI	<1.0
Histone Ab	<1.0 NEG AI	<1.0
CRP	<1.0 mg/dL	<0.40
ESR	0–20 mm/HR	16
CCP Ab IgG	0.0 to 5.0 unit: U/mL	<16
Cardiolipin Ab IgG	<20 GPLU/mL	<14
Cardiolipin Ab IgM	<20 MPL U/mL	<12
Cardiolipin Ab IgA	<22 APL U/mL	<11
Lupus anticoagulant	Not detected	Not detected
PTT-LA screen	28.0 to 43.0 secs	<31
dRVVT screen	<42.9 secs	<38
B2 glycoprotein I IgG Ab	<20 G units	<9
B2 glycoprotein I IgA Ab	<20 A units	<9
B2 glycoprotein I IgM Ab	<11 M units	<9
Triiodothyronine	76–181 ng/dL	118

^*∗*^HEp-2: human larynx epithelioma cancer (HEp-2) cell lines. ^*∗∗*^IgG ELISA: immunoglobulin G enzyme-linked immunoassay. Extractable nuclear antigen: run on enzyme-linked immunoassay (ELISA) assay kit.

**Table 3:** Leukemia/lymphoma evaluation.

Items	Results
Viability %	52%
Gate B	Lymphocytes
Marker	Percentage
CD2	72
CD3	72
CD4	43
CD5	72
CD7	64
CD8	26
CD10	2
CD11c	1
CD13	0
CD19	26
CD19+CD5+	1
CD20	26
CD23	6
CD33	0
CD34	0
CD38	63
CD45	100
CD56+CD3−	1
CD64	0
CD117	0
HLA_DR	29
Kappa CD19+	14
Lamda CD19+	11
K/L ratio	1.27

## Data Availability

Data can be obtained from the corresponding author upon request.
